# Horizontal Ridge Augmentation and Contextual Implant Placement with a Resorbable Membrane and Particulated Anorganic Bovine Bone-Derived Mineral

**DOI:** 10.1155/2019/6710340

**Published:** 2019-09-17

**Authors:** Ferdinando Attanasio, Andrea Pacifici, Amerigo Giudice, Antonella Polimeni, Luciano Pacifici

**Affiliations:** ^1^Department of Health Sciences, Magna Graecia, University of Catanzaro, Catanzaro, Italy; ^2^Department of Oral and Maxillo-Facial Science, Sapienza University of Rome, Rome 00100, Italy

## Abstract

Alveolar ridge deficiency is considered a major limitation for successful implant placement. Various approaches have been developed to horizontal augmentation of bone volume. This case report presents the medium-term results of one-stage guided bone augmentation using an anorganic bovine bone (70%) and autologous bone (30%), placed in layers, in association with resorbable collagen membrane for a subsequent implant placement. The patient presented with a localized horizontal ridge defect in the posterior zone of the jaw. The clinical and radiographic presentations, as well as relevant literature, are presented.

## 1. Introduction

Over the years, prosthetic rehabilitations supported by osteointegrated implant has become a common treatment modality in daily dental practice. After the development of the principles of osseointegration and their application to the most complex conditions, new methodologies have evolved with the aim of predicting the integration in the bone tissue [1]. With the improvement of surgical techniques, materials, and knowledge, implant therapy went from an anatomically guided procedure to a prosthetically guided procedure. In the past, surgeons used to decide to place implants where there was a sufficient amount of bone in order to ensure long-term success of osteointegrated implants. However, this procedure could lead to two types of problems: an aesthetic and a mechanical one. From a mechanical point of view, the positioning of an implant should ideally take place along the direction of the load force of the tooth or of the group of teeth to be rehabilitated in order to avoid overloading and peri-implant bone resorption's risks [2]. Thanks to the surgical techniques and the biomaterials available today, it is possible to reconstruct three-dimensional atrophic edentulous areas of the jaws thus allowing the clinician to correctly position the implants and protect themselves from failures and mechanical/prosthetic failures. GBR has been used for horizontal and vertical ridge augmentations and has demonstrated reproducible outcomes with high implant survival rates and low complication rates. Both resorbable and nonresorbable barrier membranes have proven clinical effectiveness [3–5]. Nevertheless, guided bone regeneration by means of titanium-reinforced expanded polytetrauoroethylene membrane (e-PTFE) has proven to be a successful and predictable technique for vertical and horizontal ridge augmentation both in short- and long-term studies [6–8]. However, the use of a barrier membrane is a technique-sensitive procedure, and it is not lacking in complications [9]. The most frequently reported problems involve the soft tissue; very often, the exposure of the membrane forces the operator to remove it with the possible compromise of the entire area subject to regeneration [10]. This clinical case report details the successful use of an inorganic bovine-derived bone mineral and a resorbable barrier membrane to reconstruct a severe alveolar posterior mandibulary bone defect.

## 2. Materials and Methods

**Figure 1:**
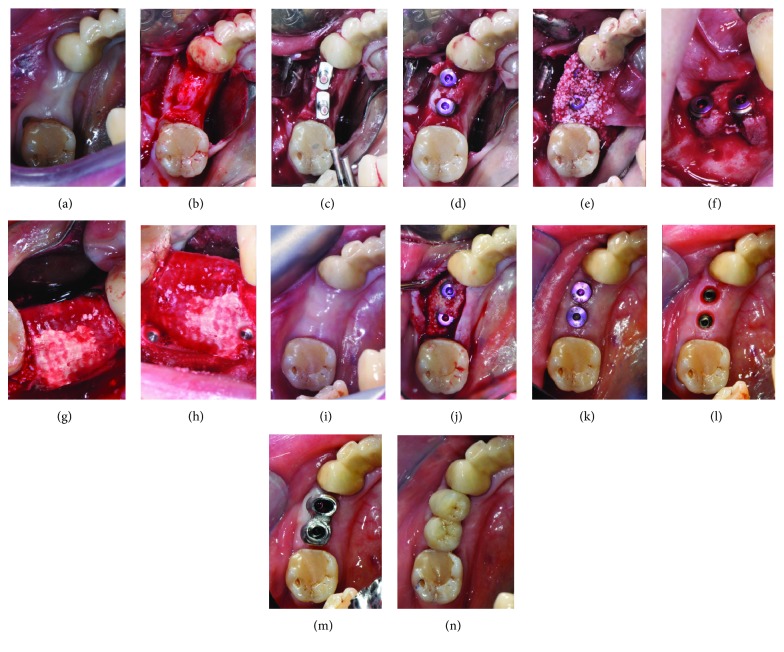
Occlusal views (a, b) of the posterior mandibular area show the thin bone crest. Occlusal view (c, d) of the two implant osteotomies and implant in place. (e) Autogenous particulate bone mixed with inorganic bovine bone-derived mineral (ABBM) in place. Buccal (f, h) and occlusal (g) views of the membrane fixed with titanium pins. Occlusal view (i) of the posterior mandibular area 6-months postsurgery. Occlusal view (j) of the regenerated bone. Second surgery performed by a lingual approach with roll flap technique that allows to increase the vestibular volume of soft tissues. (k) Healing screw in place. (l) Removal of healing screws and taking impressions. (m, n) Placement of the definitive crown (screw-retained).

A healthy 50-year-old woman presented for an evaluation of her posterior right mandibular and tooth loss in the premolar region. She reported that teeth were extracted several years previously due to a history of dentoalveolar infections. The patient was overall a healthy nonsmoker with good oral hygiene habits. Several years postextraction, the patient wanted implant therapy. As foreseen, there was a severe horizontal ridge defect, which meant the site was seriously compromised for implant reconstruction. Radiographic examination showed that the bone width in this area was not sufficient for standard implant placement. Since the patient wanted a fixed prosthesis rehabilitation with implants, the treatment plan included regeneration of the alveolar defect to ideally restore form, function, and esthetics with the immediate placement of two fixtures (Figure 1).

### 2.1. Surgical Procedure

The surgical technique selection was made in accordance to the protocol established by Urban et al. [11]. The patient was premedicated with amoxicillin+clavulanic acid (2 g) 1 hour before surgery, and then, 875 mg of amoxicillin+125 mg of clavulanic acid was administered twice a day for 1 week following surgery. The patient rinsed with a 0.20% chlorhexidine gluconate solution (Curasept, Curaden HealthCare, Italy) for 1 minute, then the skin surrounding the surgical site was disinfected. The patient presented with a thick biotype. Under local anesthesia (2% lidocaine with 1 : 80,000 epinephrine), the flap was designed to provide a clear view of the surgical area and to ensure primary tension-free closure. A full-thickness, midcrestal incision was made in the keratinized gingiva on the alveolar crest. For adequate surgical access, a divergent vertical incision was made at the mesial line angle of the mandibular right canine and another vertical incision was made at the distal line angle of the mandibular right first molar. The flap design meant that primary tension-free closure would have to be achieved over a larger dimension after the bone graft was applied to the defect. After the primary incisions, periosteal elevators were used to create a full-thickness flap beyond the mucogingival junction and at least 5 mm beyond the bone defect [12].

A periosteal releasing incision connecting the two vertical incisions was performed to achieve elasticity of the flap; the crestal cut continued 5 mm distally from it; to preserve the lingual nerve, the blade was inclined 45 degrees with the tip in the buccal direction and the external oblique ridge [13]. One implant 3,6 Ø × 11 mm long and one implant 3,6 Ø × 9 mm long OsseoSpeed EV (Astra Tech, Dentsply, Molndal, Sweden) were placed following the manufacturer's protocol in the 4.4 and 4.5 tooth site. The implants were submerged in a two-stage procedure. Given that, as planned, the procedure led to an incomplete implant incorporation into the mandibular bone matrix and proceeded to a horizontal regeneration of the defect. Autogenous bone was then harvested by the use of a manual scraper from the bone adjacent to the area to be regenerated and mixed with anorganic bovine bone-derived mineral (ABBM) (Creos Xenogain, Nobel Biocare, Goteborg, Sweden) to form a composite bone graft. Multiple decortical holes were made in the bony bed using a small round burr in order to reveal the medullary space. The correct size of a collagen membrane (T-BARRIER, B&B Dental Implant Company, Bologna, Italy) was trimmed allowing for graft volume. The membrane was positioned on the lingual side using a titanium pin. The composite bone graft was put into the defect, and the membrane was folded over and positioned using the other two titanium pins on the vestibular side. Using an expanded polyester suture (Ethibond, Ethicon, Somerville, NJ), the flap was then stitched in two layers. Horizontal mattress stitches were made 4 mm from the incision line followed by single interrupted stitches to close the edges of the flap allowing a minimum of a 4 mm deep connective tissue layer between the membrane and the oral epithelium. Using single interrupted stitches, the vertical incisions were closed. These single stitches were removed 10 to 14 days after surgery while the mattress stitches were taken out after 3 weeks. In addition to the pre- and postoperative antibiotics described above, an anti-inflammatory medication (ibuprofen 600 mg) immediately after the surgical intervention and thereafter three times a day for 1 week following surgery was prescribed. Chemical plaque control using a 0.20% chlorhexidine gluconate solution was used twice a day from 24 hours postsurgery until the time of suture removal. After 6 months, an envelope flap was created for the second surgical stage which consisted in a paracrestal incision 3 mm lingual from the crest. The keratinized gingiva was positioned and stitched on the buccal aspect of the healing abutments. This uncovery procedure ensured good quality keratinized tissue all around the implants, thus improving not only the aesthetics of the area but also the function, preventing unpleasant accumulations of food for the patient in that area given by the concavity of the site. The implants were restored with screw-retained implant-supported porcelain fused-to-metal crowns, and all the laboratory steps followed a completely digital workflow.

## 3. Results and Discussion

**Figure 2:**
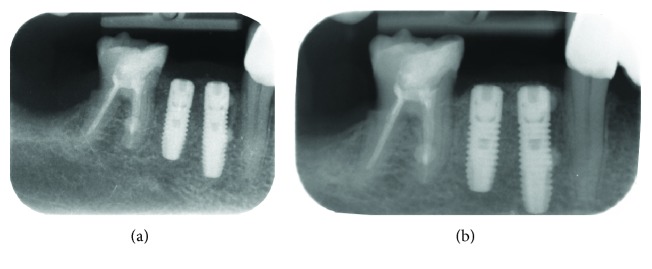
Radiographic exams. (a) Rx right after implant surgery: it highlights the correct implant axis, the connection with the proximal roots, and the implant's fitness under the bone crest. (b) Rx control after 6 months: it shows the physiological bone remodeling around the implant's head.

The implants remained in function, and the patient did not complain of foreign body sensation, pain, or dysesthesia. Intraoral examinations showed healthy peri-implant mucosa without suppuration, swelling, or redness at any implant site. Radiologic examination showed the first bone-implant contact was located near the first implant thread (Figure 2).

Guided bone regeneration is still a daunting element in the dental implant treatment protocol.

In order to arrive at a good long-term scenario for osteointegrated implants, there has to be enough bone volume at the implantation sites. To replace the lost bone and to permit the complete implant's integration and maintained during functional loading [14–16], diverse approaches, such as bone-grafting techniques, alveolar distraction, and guided bone regeneration, have been practiced. Guided bone regeneration is thought to be the most commonly used alveolar bone's reconstructing method, also used to treat peri-implant bone deficiencies [17–20]. Research indicates that survival rates of implants positioned in the sites enlarged by GBR are comparable to those found for implants in pristine sites [16, 21–23]. In particular, the implant survival rate in the sites that have received GBR procedures through the use of xenogenic materials is greater compared to the sites that have received GBR procedures through the insertion of autogenous bone blocks; in a prospective study by Meloni et al. (2019), data confirm the 1-year results allowing for the use of collagen resorbable membrane in GBR procedures for horizontal ridge augmentation; although the two-stage approach needs a longer time before prosthesis delivery, this technique seems to be safe and predictable for large reconstruction and can be applied in daily practice [24].

The survival rate of implants positioned in enlarged sites oscillated between 79 and 100%, and the bulk of research showed a survival rate of over 90% after at least one year of utility [25]. An essential element in the treatment is the membrane and its various materials and adaptations used for GBR. The membrane used for GBR therapy should have biocompatibility, cell-occlusion properties, integration by the host tissues, clinical manageability, the ability to make space, and adequate mechanical and physical properties.

Nonresorbable membranes, for the most part polytetrafluoroethylene (PTFE) in its expanded form (e-PTFE), made up the first generation of barrier membranes. These membrane types generally showed biocompatibility and space-making capability [26], but nonresorbable membranes require another surgical operation to remove the membrane. Later, a second generation of resorbable membranes was developed, and their use became widespread in diverse clinical situations. Of late, work has been carried out to develop a further generation of membranes by using naturally derived membranes or employing principles of tissue engineering during the preparation of the membrane [27, 28]. In addition, the use of membranes in the defect, along with bone grafts and substitute materials, is regularly employed to give a structural support to the defect site and to advocate the intrinsic regenerative potential of the host tissue. Horizontal ridge augmentation has been illustrated using a variety of different techniques and materials [29]. In the case in question, we used a resorbable collagen membrane so as to avoid some of the drawbacks when using nonresorbable membranes, like the necessity for a second surgical procedure to remove the membrane combined with the risk of losing more of the regenerated bone due to flap reflection [30]. Moreover, the conclusion of an in vitro study which compared resorbable and nonresorbable membranes was that bioabsorbable membranes, particularly collagen and hyaluronic acid, may promote bone regeneration through their activity on osteoblasts which suggests that bioabsorbable membranes could be more apt than nonresorbable membranes because they encourage the regeneration and repair of bones [31].

Since they are not rigid, most bioabsorbable membranes have to be combined with a graft material to ensure space maintenance when used for bone augmentation; otherwise, barrier membranes could get compressed into the space of a bony defect by the overlying soft tissue during the healing process [29]. Thus, a blend of particulate of autogenous and xenogeneic grafts was used together with the membrane. Autograft is deemed the gold standard for the majority of craniofacial bone grafting as well as for the treatment of various dental implant-related defects. On the other hand, autografts have known weaknesses which include donor site morbidity, potential resorption, size mismatch, and insufficient quantity of graft material.

The most important complication with the autologous bone is certainly the comorbidity associated with the presence of a donor site which needs a second surgical site. The complications described in the literature with this type of material appear to be chronic pain in a range of 2.5% from 8% of cases, dysesthesia in 6% of cases, or infection in 2% of cases. An alternative to autologous bone is the use of allogenic, but some risk of disease transmission can exists. Moreover, the high cost of such materials should be considered. Another alternative to autograft could be the use of xenogenic bone [32]. In our case, none of these potential complications came up and healing was uneventful during the follow-up period. We were able to overcome the lack of titanium reinforcement for the resorbable membrane by securely fixing the membrane on both the lingual/palatal and the vestibular side. In this way, the graft material is immobilized permitting the formation of the required quantity of bone.

## 4. Conclusions

Within the confines of this case report, we can consider the GBR technique to be successful in the preprosthetic surgical treatment of horizontally deficient alveolar ridges, allowing in these cases the execution of prosthetically guided implant-supported oral rehabilitations. Anyway, the use of a barrier membrane is a technique-sensitive procedure and it is not lacking in complications. The GBR technique using autogenous bone, with ABBM, and a resorbable barrier membrane fixed with titanium pins, allows the patient to save further surgery and therefore reduces the comorbidity of the procedure and the risk of complications such as the loss of regenerated tissue. The regenerated bone showed to provide good osteointegration of the dental implants. We would stress that the study in question is a case report and so conclusions cannot be generalized. Further-reaching, long-term studies will be necessary to back up this premise so as to apply this technique for other clinical cases.
